# Understanding Sickle cell disease: Causes, symptoms, and treatment options

**DOI:** 10.1097/MD.0000000000035237

**Published:** 2023-09-22

**Authors:** Chukwuka Elendu, Dependable C. Amaechi, Chisom E. Alakwe-Ojimba, Tochi C. Elendu, Rhoda C. Elendu, Chiagozie P. Ayabazu, Titilayo O. Aina, Ooreofe Aborisade, Joseph S. Adenikinju

**Affiliations:** a Federal University Teaching Hospital, Owerri, Nigeria; b Igbinedion University Okada, Okada, Nigeria; c Chukwuemeka Odumegwu Ojukwu University, Anambra State, Nigeria; d Imo State University, Owerri, Nigeria; e van Horbachevsky Ternopil National Medical University, Ternopil, Ukraine; f Babcock University, Ogun State, Nigeria; g Olabisi Onabanjo University Teaching Hospital, Sagamu, Nigeria; h Northwick Park Hospital London Northwest NHS Trust, Harrow, London.

**Keywords:** acute chest syndrome, disease-modifying therapies, hematopoietic stem cell transplantation, Sickle cell disease, stroke, vaso-occlusive crisis

## Abstract

Sickle cell disease (SCD) is a hereditary blood disorder characterized by the production of abnormal hemoglobin molecules that cause red blood cells to take on a crescent or sickle shape. This condition affects millions of people worldwide, particularly those of African, Mediterranean, Middle Eastern, and South Asian descent. This paper aims to provide an overview of SCD by exploring its causes, symptoms, and available treatment options.

The primary cause of SCD is a mutation in the gene responsible for producing hemoglobin, the protein that carries oxygen in red blood cells. This mutation has abnormal hemoglobin called hemoglobin S, which causes red blood cells to become stiff and sticky, leading to various health complications. Patients with SCD may experience recurrent pain, fatigue, anemia, and increased infection susceptibility.

Treatment options for SCD focus on managing symptoms and preventing complications. This includes pain management with analgesics, hydration, and blood transfusions to improve oxygen delivery. Hydroxyurea, a medication that increases the production of fetal hemoglobin, is commonly used to reduce the frequency and severity of pain crises. Additionally, bone marrow or stem cell transplants can cure select individuals with severe SCD.

Finally, understanding the causes, symptoms, and treatment options for SCD is crucial for healthcare professionals, patients, and their families. It enables early diagnosis, effective symptom management, and improved quality of life for individuals with this chronic condition.

## 1. Introduction and background

Sickle cell disease (SCD) is a hereditary blood disorder characterized by the production of abnormal hemoglobin molecules that cause red blood cells to take on a crescent or sickle shape. This condition affects millions of people worldwide, particularly those of African, Mediterranean, Middle Eastern, and South Asian descent.^[1]^ SCD significantly impacts the health and quality of life of individuals affected by the disease. Therefore, understanding the causes, symptoms, and treatment options is essential for healthcare professionals, patients, and their families.

The primary cause of SCD is a mutation in the gene responsible for producing hemoglobin, known as the beta-globin gene (HBB) gene.^[2]^ This gene mutation produces abnormal hemoglobin called hemoglobin S (HbS), which differs from normal adult hemoglobin (HbA) in its molecular structure. HbS causes red blood cells to become stiff and sticky, resulting in their deformation and reduced ability to flow through small blood vessels.^[3]^ The altered shape of red blood cells contributes to various health complications associated with SCD.

The clinical manifestations of SCD are diverse and can vary in severity among individuals. The hallmark symptom is recurrent episodes of severe pain, known as vaso-occlusive crises, which result from the obstruction of blood flow in small vessels by sickled red blood cells.^[4]^ Fatigue, anemia, and increased susceptibility to infections are also common symptoms of SCD.^[5]^ These manifestations can significantly impact the daily lives of individuals with SCD, affecting their physical and psychosocial well-being.

Treatment options for SCD aim to manage symptoms, prevent complications, and improve the quality of life for patients. Pain management is critical and often involves using analgesics, including opioids, nonsteroidal anti-inflammatory drugs, and adjuvant therapies.^[5]^ Adequate hydration, both orally and intravenously, is essential to maintain proper blood flow and prevent the sickling of red blood cells.^[6]^ In some instances, regular blood transfusions may be required to improve oxygen delivery to tissues and reduce the risk of complications such as stroke.^[7]^

Hydroxyurea, a medication that increases the production of fetal hemoglobin (HbF), has proven to be effective in reducing the frequency and severity of pain crises in SCD.^[8]^ HbF inhibits red blood cells’ sickling, improving their survival and overall clinical outcomes. Furthermore, bone marrow or stem cell transplants can offer a potential cure for select individuals with severe SCD, although this option is limited by donor availability and associated risks.^[9]^

Understanding the causes, symptoms, and treatment options for SCD is crucial for healthcare professionals to provide accurate diagnosis, effective symptom management, and appropriate counseling for patients and their families. Further research and advancements in gene therapies hold promise for future treatments, offering hope for a better future for SCD patients.

## 2. Objective of study

This study aims to provide a comprehensive overview of SCD by examining its causes, symptoms, and available treatment options. The study aims to consolidate knowledge on SCD, synthesizing information from reputable sources and scientific literature. By achieving this objective, the study seeks to enhance understanding and awareness of SCD among healthcare professionals, patients, and their families.

Specifically, the study aims to:

Identify the primary cause of SCD, focusing on the genetic mutation in the HBB gene responsible for abnormal hemoglobin production.

Explore the symptoms and clinical manifestations of SCD, including recurrent pain crises, anemia, and increased susceptibility to infections.

Investigate the various treatment options for managing SCD, such as pain management strategies, hydration techniques, blood transfusions, and medications like hydroxyurea.

Assess the effectiveness and limitations of current treatment approaches, highlighting their impact on symptom relief and overall quality of life for individuals with SCD.

Discuss emerging research and potential future treatment options, such as gene therapies and stem cell transplants, which offer hope for improved outcomes and possible cures for SCD.

By addressing these objectives, this study aims to contribute to the knowledge surrounding SCD and provide valuable information for healthcare professionals, patients, and their families. The findings of this study can inform clinical practice, enhance patient care, and promote ongoing research efforts to improve the management and treatment of SCD.

## 3. Review

### 3.1. Methodology

This study systematically gathered and analyzed information on SCD causes, symptoms, and treatment options. The methodology involved a comprehensive review of reputable sources, including scientific literature, peer-reviewed journals, and authoritative medical websites. The following steps were undertaken:

#### 3.1.1. Literature search.

A thorough search was conducted using online databases, such as PubMed, Google Scholar, and Medline, to identify relevant articles, reviews, and studies related to SCD. Keywords used in the search included “sickle cell disease,” “sickle cell anemia,” “causes,” “symptoms,” and “treatment options.”

#### 3.1.2. Inclusion and exclusion criteria.

The search results were screened based on predefined inclusion and exclusion criteria. Only articles written in English and published within the last ten years were considered. Studies on human subjects, clinical trials, and reviews providing comprehensive insights into SCD causes, symptoms, and treatment options were included.

#### 3.1.3. Data extraction.

Pertinent data and information were extracted from the selected articles. This included details on the genetic basis of SCD, the pathophysiology of the disease, common symptoms and complications, and various treatment modalities. Key findings, statistics, and clinical recommendations were recorded.

#### 3.1.4. Data analysis.

The extracted data were analyzed and organized thematically. Similarities and patterns in the findings were identified, and key concepts related to SCD causes, symptoms, and treatment options were synthesized. Data were then categorized into subtopics for a coherent presentation.

#### 3.1.5. Citation and referencing.

All sources used in the study were adequately cited and referenced. The references were formatted according to the appropriate citation style (e.g., American Psychological Association and Modern Language Association) to ensure accuracy and consistency.

#### 3.1.6. Manuscript composition.

The findings and insights from the analysis were synthesized and used to construct the study abstract, introduction, objectives, and methodology sections.

By employing this methodology, this study ensured a rigorous and systematic approach to gathering and analyzing relevant information on SCD causes, symptoms, and treatment options. The utilization of reputable sources and adherence to inclusion and exclusion criteria enhanced the validity and reliability of the findings.

### 3.2. Definition and epidemiology

SCD is a globally prevalent hereditary blood disorder with a significant impact on affected individuals and healthcare systems. The epidemiology of SCD varies across different regions and populations. This section provides a detailed overview of the epidemiological characteristics of SCD, including its global distribution, prevalence, and people at risk.

SCD primarily affects populations with ancestral origins in regions where malaria is or has been endemic, including sub-Saharan Africa, the Mediterranean, the Middle East, and parts of India and Southeast Asia.^[10]^ These regions are characterized by a higher prevalence of the sickle cell trait (carrying 1 copy of the mutated gene) due to its protective effect against malaria. Consequently, the incidence of SCD is highest in these areas.

According to estimates, SCD affects millions of individuals worldwide. In sub-Saharan Africa alone, it is estimated that over 70% of all SCD cases occur, with approximately 300,000 affected infants born each year.^[11]^ In the United States, SCD primarily affects individuals of African descent, with an estimated prevalence of 1 in 365 African American births.^[12]^ Other populations with a higher prevalence of SCD include individuals of Hispanic, Mediterranean, Middle Eastern, and South Asian descent.^[13]^

The prevalence of SCD varies within populations and across geographical regions. In Africa, the prevalence can range from 10% to 40% in certain tribal groups.^[14]^ The prevalence varies among different states in the United States, with higher rates observed in regions with larger African American populations.^[15]^ In some areas of the Mediterranean, such as Saudi Arabia, the prevalence of SCD reaches up to 4%.^[16]^

The impact of SCD extends beyond prevalence rates, affecting morbidity, mortality, and healthcare utilization. Individuals with SCD face a range of health complications, including acute and chronic pain crises, increased susceptibility to infections, organ damage, and reduced life expectancy.^[17]^ These complications significantly burden healthcare systems, with increased hospitalizations, emergency department visits, and the need for specialized care.

Various factors, including genetic inheritance patterns, geographical location, and socioeconomic factors, influence the epidemiology of SCD. Genetic counseling and carrier screening programs are crucial in identifying individuals at risk of having children with SCD and enabling informed family planning decisions.

Understanding the epidemiology of SCD is essential for public health planning, resource allocation, and the development of effective prevention and management strategies. It helps healthcare professionals and policymakers identify at-risk populations, implement targeted interventions, and improve access to comprehensive care for individuals affected by SCD.

### 3.3. Pathophysiology

SCD is characterized by a complex pathophysiology involving the abnormal sickling of red blood cells, altered blood rheology, and subsequent tissue damage. The pathophysiological processes in SCD are primarily driven by the structural and functional changes in hemoglobin and the resultant sickle-shaped red blood cells. This section provides a detailed overview of the critical mechanisms underlying the pathophysiology of SCD.

HbS polymerization: The primary abnormality in SCD lies in substituting glutamic acid with valine at the sixth position of the beta-globin chain, leading to the formation of abnormal hemoglobin known as HbS. Under certain conditions, such as low oxygen tension or dehydration, HbS undergoes polymerization, forming long, stiff polymers within the red blood cells.^[3]^

Sickling of red blood cells: Polymerization of HbS results in the deformation of red blood cells into a sickle shape. The sickled red blood cells are rigid, less deformable, and prone to hemolysis. These cells cannot flow smoothly through blood vessels, leading to vaso-occlusion and tissue ischemia.^[18]^

Vaso-occlusion: Sickled red blood cells can adhere to endothelial cells and other sickled cells, forming aggregates that obstruct blood flow in small blood vessels. This vaso-occlusion contributes to tissue ischemia, resulting in acute pain crises, organ damage, and increased susceptibility to infections.^[19]^

Increased blood viscosity: The presence of sickled red blood cells and increased levels of circulating inflammatory cells and plasma proteins leads to increased blood viscosity in individuals with SCD. This elevated viscosity further contributes to impaired blood flow, vaso-occlusion, and tissue damage.^[20]^

Oxidative stress and inflammation: SCD is associated with increased oxidative stress due to the presence of free heme and iron released from hemolysis. Oxidative stress triggers inflammatory responses, activation of endothelial cells, and adhesion of sickled red blood cells to the vascular endothelium. This inflammatory cascade further promotes vaso-occlusion and endothelial dysfunction.^[21]^

Endothelial dysfunction: The interactions between sickled red blood cells and endothelial cells lead to endothelial activation and dysfunction. Endothelial dysfunction produces pro-inflammatory mediators, vasoconstriction, and increased adherence of sickled cells to the endothelium, exacerbating vaso-occlusion and tissue damage.^[22]^

Ischemia-reperfusion injury: Repeated episodes of vaso-occlusion followed by reperfusion during blood flow restoration can contribute to ischemia-reperfusion injury. This process involves the generation of reactive oxygen species, inflammation, and tissue damage, further exacerbating the pathophysiological consequences of SCD.^[23]^

Understanding the underlying pathophysiological mechanisms of SCD is crucial for developing targeted therapeutic interventions. Current treatment approaches aim to prevent or mitigate vaso-occlusive crises, manage complications, and improve the overall quality of life for individuals with SCD.

### 3.4. Causes

SCD is primarily caused by a genetic mutation affecting hemoglobin, the protein responsible for carrying oxygen in red blood cells. The underlying cause of SCD lies in a point mutation in the HBB on chromosome 11, resulting in the production of abnormal hemoglobin known as HbS.^[1]^

The specific mutation involves a substitution of a single nucleotide, where adenine is replaced by thymine, leading to the substitution of glutamic acid with valine at the sixth position of the beta-globin chain.^[2]^ This alteration affects the structure and function of hemoglobin, causing it to polymerize under certain conditions, such as low oxygen tension or dehydration.

The polymerization of HbS leads to the deformation of red blood cells into a characteristic sickle shape, which is rigid and prone to hemolysis. The sickled red blood cells cannot flow smoothly through blood vessels, leading to vaso-occlusion, tissue ischemia, and subsequent organ damage.^[3]^

SCD follows an autosomal recessive inheritance pattern, meaning an individual must inherit 2 copies of the mutated gene (one from each parent) to develop the disease. Individuals who inherit 1 copy of the mutated gene and 1 normal gene have the sickle cell trait and are generally asymptomatic but can pass the trait on to their offspring.

The prevalence of SCD is higher in populations with a historical association with malaria, as the sickle cell trait provides some protection against severe forms of malaria infection. As a result, SCD is more commonly found in regions where malaria is or has been endemic, such as sub-Saharan Africa, the Mediterranean, the Middle East, and parts of India and Southeast Asia.^[1]^

Understanding the genetic basis and underlying cause of SCD has paved the way for advancements in genetic counseling and carrier screening programs. These programs help identify individuals at risk of having children with SCD, enabling informed family planning decisions and providing supportive care for affected individuals and their families.

### 3.5. Symptoms

SCD is characterized by a wide range of symptoms that can vary in severity and presentation among individuals. The symptoms primarily arise due to the abnormal sickling of red blood cells and subsequent complications. This section provides a detailed overview of the common symptoms associated with SCD.

#### Pain crises:

Recurrent episodes of severe pain, known as vaso-occlusive crises or pain crises, are a hallmark of SCD. These painful episodes occur due to the blockage of blood vessels by sickled red blood cells, leading to tissue ischemia and inflammation. Pain can occur in various body parts, including the chest, abdomen, bones, and joints.^[5]^

#### Anemia:

SCD causes chronic hemolytic anemia, characterized by the destruction of red blood cells at an accelerated rate. Anemia can result in fatigue, weakness, paleness, and shortness of breath.^[5]^

#### Infections:

Individuals with SCD are more susceptible to infections, particularly bacterial infections, due to functional asplenia (loss of spleen) and impaired immune function. Common infections include pneumonia, urinary tract infections, and bacterial sepsis.^[24]^

#### Acute chest syndrome:

This is a severe complication of SCD characterized by chest pain, fever, cough, and difficulty breathing. It is often caused by infection, pulmonary infarction, or fat embolism and can be life-threatening.^[25]^

#### Delayed growth and development:

Children with SCD may experience delayed growth and development compared to their peers. Chronic anemia, nutrient deficiencies, and the impact of recurrent pain crises on daily activities can contribute to growth and developmental challenges.^[26]^

#### Stroke:

SCD increases the risk of stroke, particularly in children. Obstruction of blood vessels in the brain by sickled red blood cells can lead to ischemic stroke. The risk factors for stroke include a history of previous transient ischemic attacks and abnormal blood flow detected by transcranial Doppler ultrasonography.^[27]^

#### Organ damage:

SCD can lead to long-term organ damage. Organs commonly affected include the spleen (leading to functional asplenia), kidneys (resulting in renal dysfunction), eyes (causing retinopathy), and bones (increasing the risk of avascular necrosis).^[5]^

It is important to note that the severity and frequency of symptoms can vary among individuals with SCD. Some individuals may experience milder symptoms and better quality of life, while others may have more frequent and severe complications.

Prompt medical attention, comprehensive care, and early intervention are essential in managing the symptoms of SCD and minimizing complications. Regular monitoring, pain management strategies, preventive antibiotics, vaccinations, and supportive care constitute SCD management cornerstone.

### 3.6. Investigations and diagnosis

The diagnosis of SCD involves a combination of clinical evaluation, laboratory tests, and genetic testing. The aim is to identify the presence of abnormal HbS and assess the extent of the disease. This section provides a detailed overview of the investigations and diagnostic approaches used for SCD.

#### Complete blood count:

A total blood count helps assess the levels of hemoglobin, red blood cells, and other cell types. Due to chronic hemolysis, individuals with SCD typically exhibit a lower hemoglobin level and a higher reticulocyte count.^[1]^

#### Hemoglobin electrophoresis:

Hemoglobin electrophoresis is a crucial diagnostic test that identifies the presence of abnormal hemoglobin variants. It separates different hemoglobin types based on their electrical charge. It provides information about the relative quantities of HbS and other hemoglobin types, such as hemoglobin A (HbA) and hemoglobin F (HbF).^[28]^

#### Sickledex/solubility test:

The Sickledex or solubility test is a quick screening test that detects the presence of HbS in a blood sample. It relies on the insolubility of HbS under certain conditions, leading to the formation of sickle-shaped cells. However, this test is less specific than hemoglobin electrophoresis and may require confirmation with additional tests.^[18]^

#### Hemoglobin high-performance liquid chromatography:

Hemoglobin high-performance liquid chromatography is a more advanced technique for accurately quantifying and identifying different hemoglobin variants. It provides a detailed analysis of the relative proportions of HbS, HbA, HbF, and other hemoglobin types.^[29]^

#### Genetic testing:

Genetic testing is performed to confirm the diagnosis of SCD and to identify specific mutations in the HBB. This can involve DNA analysis, including polymerase chain reaction, gene sequencing, and other molecular techniques, to detect the presence of the HbS mutation and potentially identify genetic variants.^[30]^

#### Newborn screening:

Newborn screening programs are implemented in many countries to identify infants with SCD early on. This typically involves testing a blood sample from newborns to detect abnormal hemoglobin patterns. Early diagnosis through newborn screening enables early intervention and comprehensive care for affected infants.^[31]^

It is important to note that a comprehensive evaluation of individuals suspected of SCD includes a detailed medical history, physical examination, and assessment of clinical symptoms. Additional tests, such as imaging studies (e.g., ultrasound, magnetic resonance imaging) and specialized evaluations (e.g., transcranial Doppler ultrasound for stroke risk assessment), may be performed to evaluate organ involvement and monitor disease complications.^[32]^

### 3.7. Treatment

SCD management aims to alleviate symptoms, prevent complications, and improve the overall quality of life for individuals with the condition. The treatment of SCD involves a multidisciplinary approach that addresses various aspects of the disease. This section provides a detailed overview of the treatment options commonly employed for SCD.

#### 3.7.1. Supportive care.

##### Pain management:

Acute pain crises, a hallmark of SCD, are managed with analgesic medications such as nonsteroidal anti-inflammatory drugs, opioids, and patient-controlled analgesia. Non-pharmacological approaches may also be employed, including heat therapy, relaxation, and distraction techniques.^[33]^

##### Hydration:

Adequate hydration helps prevent vaso-occlusive crises. Patients are encouraged to drink plenty of fluids, particularly during increased risk, such as infections or exposure to extreme temperatures.

##### Blood transfusions:

Red blood cell transfusions may be administered in specific situations, such as severe anemia, acute chest syndrome (ACA), or stroke. Transfusions help increase the oxygen-carrying capacity of the blood and reduce the percentage of sickled cells.^[34]^

#### 3.7.2. Disease-modifying therapies.

##### Hydroxyurea:

Hydroxyurea is a medication that stimulates the production of HbF, which inhibits the sickling of red blood cells. It has been shown to reduce the frequency of pain crises, ACA, and hospitalizations. Hydroxyurea is generally well-tolerated, but regular monitoring of blood counts and liver function is required.^[8]^

##### Hematopoietic stem cell transplantation (HSCT):

HSCT is a potentially curative option for eligible patients, particularly children with severe SCD. It involves replacing the diseased bone marrow with healthy stem cells from a compatible donor. HSCT carries significant risks and requires careful consideration and evaluation of eligibility.^[35]^

#### 3.7.3. Complication-specific treatments.

##### Antibiotics:

Prophylactic antibiotics, such as penicillin, are often prescribed to children with SCD to prevent infections, particularly those caused by Streptococcus pneumonia. Vaccinations against common bacterial infections, including pneumococcus and meningococcus, are also recommended.^[34]^

##### Transfusion therapy:

Regular blood transfusions may be indicated in individuals with SCD complications such as stroke or recurrent ACA. Transfusions can help reduce the risk of these complications by decreasing the percentage of sickled cells and increasing the oxygen-carrying capacity of the blood.^[34]^

##### Pulmonary hypertension management:

In individuals with SCD-related pulmonary hypertension, targeted therapies such as endothelin receptor antagonists, phosphodiesterase-5 inhibitors, and prostacyclin analogs may be prescribed to improve symptoms and slow disease progression.^[36]^

#### 3.7.4. Supportive measures.

##### Comprehensive care:

Individuals with SCD benefit from comprehensive care programs that provide regular medical follow-up, psychosocial support, educational resources, and genetic counseling. These programs help individuals and their families manage the disease effectively and improve their quality of life.^[29]^

##### Pain crisis prevention:

Educating individuals with SCD about pain crisis triggers, hydration, and early recognition of symptoms can help prevent pain crises. Prompt treatment of underlying infections and avoiding exposure to extreme temperatures are essential preventive measures.^[37]^

It is crucial for individuals with SCD to receive regular medical care from healthcare providers experienced in managing the condition. Treatment plans should be individualized based on the severity of the disease, the presence of complications, and each patient specific needs.

### 3.8. Complications

SCD has many complications that can affect multiple organ systems. These complications arise due to sickle-shaped red blood cells’ abnormal shape and function, leading to vaso-occlusion, tissue ischemia, and chronic hemolysis. This section provides a detailed overview of the common complications seen in SCD.

#### Vaso-occlusive crisis:

Vaso-occlusive crisis is the hallmark complication of SCD and is characterized by the sudden onset of severe pain, often affecting the bones, joints, and abdomen. Vaso-occlusion occurs when sickled red blood cells block blood vessels, leading to tissue ischemia, organ damage, and intense pain.^[24]^

#### Acute chest syndrome (ACS):

ACS is a potentially life-threatening complication characterized by fever, chest pain, cough, and shortness of breath. It results from the obstruction of pulmonary blood vessels by sickled red blood cells, leading to lung tissue damage and impaired gas exchange. ACS is a common cause of hospitalization in individuals with SCD.^[25]^

Stroke can occur in individuals with SCD, particularly children, due to the occlusion of blood vessels supplying the brain. Silent cerebral infarctions are common and can lead to cognitive impairments and neurodevelopmental problems. Transcranial Doppler ultrasound is used to identify children at high risk of stroke.^[38]^

#### Chronic anemia:

Chronic hemolysis and the destruction of sickled red blood cells result in chronic anemia in individuals with SCD. Anemia can cause fatigue, weakness, and decreased exercise tolerance. Regularly monitoring hemoglobin levels and iron status is essential for managing anemia.^[35]^

#### Infections:

Individuals with SCD are more susceptible to infections, particularly those caused by encapsulated bacteria such as Streptococcus pneumoniae and Haemophilus influenza. Infections can range from mild to severe, including pneumonia, meningitis, and osteomyelitis. Vaccination against common bacterial pathogens is essential.^[39]^

#### Organ damage:

SCD can affect various organs, leading to long-term complications:

#### Renal complications:

SCD-associated nephropathy can result in kidney damage and impaired kidney function, leading to chronic kidney disease and renal replacement therapy.^[40]^

#### Ocular complications:

SCD can cause retinopathy, resulting in visual impairment and blindness. Regular eye examinations are necessary to monitor for retinal changes.^[41]^

#### Priapism:

Prolonged and painful penile erection, known as priapism, can occur in males with SCD. Priapism requires immediate medical attention to prevent permanent damage to the penis.^[42]^

#### Gallbladder disease:

SCD increases the risk of gallstones and cholecystitis due to the precipitation of bilirubin in the gallbladder. Surgical removal of the gallbladder may be necessary in severe cases.^[43]^

Individuals with SCD need comprehensive medical care, including regular monitoring, preventive measures, and early intervention to manage and prevent complications.

## 4. Conclusion

SCD is a complex genetic disorder affecting millions worldwide. This debilitating condition is characterized by the abnormal shape of red blood cells, leading to a wide range of complications and significant morbidity. Understanding the causes, symptoms, and treatment options for SCD is crucial in improving the quality of life for individuals with the disease.

The causes of SCD are rooted in genetic mutations that result in the production of abnormal hemoglobin. These mutations lead to the formation of sickle-shaped red blood cells that are prone to vaso-occlusion, tissue ischemia, and chronic hemolysis. This process sets the stage for the numerous complications associated with SCD.

The symptoms of SCD can vary in severity and affect multiple organ systems. Vaso-occlusive crises, ACA, stroke, chronic anemia, and infections are some common manifestations of the disease. Prompt recognition and management of these symptoms are essential in preventing further complications and improving outcomes.

Diagnosing SCD involves a combination of laboratory tests, including hemoglobin electrophoresis and genetic testing. Early and accurate diagnosis enables appropriate interventions and the initiation of disease-modifying therapies such as hydroxyurea. Comprehensive care programs that provide regular medical follow-up, psychosocial support, and educational resources are crucial in managing the disease effectively.

Treatment of SCD focuses on supportive care, disease-modifying therapies, and addressing specific complications. Pain management, hydration, blood transfusions, and prophylactic antibiotics are employed to alleviate symptoms and prevent complications. Disease-modifying therapies like hydroxyurea and hematopoietic stem cell transplantation offer potential benefits in reducing the frequency of crises and providing a curative option, respectively.

While significant progress has been made in understanding and managing SCD, much work remains to be done. Further research is needed to advance our knowledge of the pathophysiology, identify novel treatment targets, and improve the overall care of individuals with SCD. By continuing to raise awareness, supporting research efforts, and providing comprehensive care, we can strive to enhance the quality of life for individuals living with SCD and ultimately work towards finding a cure.

## Author contributions

**Conceptualization:** Chukwuka Elendu, Dependable C. Amaechi, Tochi C. Elendu, Rhoda C. Elendu, Chiagozie P. Ayabazu.

**Data curation:** Chukwuka Elendu, Dependable C. Amaechi, Tochi C. Elendu, Chiagozie P. Ayabazu.

**Formal analysis:** Chukwuka Elendu, Dependable C. Amaechi, Tochi C. Elendu, Chiagozie P. Ayabazu.

**Investigation:** Chukwuka Elendu, Dependable C. Amaechi, Tochi C. Elendu, Chiagozie P. Ayabazu.

**Methodology:** Chukwuka Elendu, Dependable C. Amaechi, Chisom E. Alakwe-Ojimba, Tochi C. Elendu, Chiagozie P. Ayabazu, Ooreofe Aborisade, Joseph S. Adenikinju.

**Project administration:** Chukwuka Elendu, Dependable C. Amaechi, Tochi C. Elendu, Chiagozie P. Ayabazu.

**Resources:** Chukwuka Elendu, Dependable C. Amaechi, Tochi C. Elendu, Chiagozie P. Ayabazu.

**Software:** Chukwuka Elendu, Dependable C. Amaechi, Tochi C. Elendu, Chiagozie P. Ayabazu.

**Supervision:** Chukwuka Elendu, Dependable C. Amaechi, Chisom E. Alakwe-Ojimba, Tochi C. Elendu, Chiagozie P. Ayabazu.

**Validation:** Chukwuka Elendu, Dependable C. Amaechi, Tochi C. Elendu, Chiagozie P. Ayabazu.

**Visualization:** Chukwuka Elendu, Dependable C. Amaechi, Tochi C. Elendu, Chiagozie P. Ayabazu.

**Writing – original draft:** Chukwuka Elendu, Dependable C. Amaechi, Tochi C. Elendu, Chiagozie P. Ayabazu.

**Writing – review & editing:** Chukwuka Elendu, Dependable C. Amaechi, Chisom E. Alakwe-Ojimba, Tochi C. Elendu, Rhoda C. Elendu, Chiagozie P. Ayabazu, Titilayo O. Aina, Ooreofe Aborisade, Joseph S. Adenikinju.
